# ADOR zeolite with 12 × 8 × 8-ring pores derived from IWR germanosilicate†

**DOI:** 10.1039/d3ta06161b

**Published:** 2023-11-29

**Authors:** Qiudi Yue, Valeryia Kasneryk, Michal Mazur, Sarra Abdi, Yong Zhou, Paul S. Wheatley, Russell E. Morris, Jiří Čejka, Mariya Shamzhy, Maksym Opanasenko

**Affiliations:** a Department of Physical and Macromolecular Chemistry, Faculty of Science, Charles University Hlavova 8 128 43 Prague 2 Czech Republic mariya.shamzhy@natur.cuni.cz; b EaStCHEM School of Chemistry, University of St Andrews St Andrews KY16 9ST UK

## Abstract

Zeolites have been well known for decades as catalytic materials and adsorbents and are traditionally prepared using the bottom-up synthesis method. Although it was productive for more than 250 zeolite frameworks, the conventional solvothermal synthesis approach provided limited control over the structural characteristics of the formed materials. In turn, the discovery and development of the Assembly-Disassembly-Organization-Reassembly (ADOR) strategy for the regioselective manipulation of germanosilicates enabled the synthesis of previously unattainable zeolites with predefined structures. To date, the family tree of ADOR materials has included the topological branches of UTL, UOV, IWW, *CTH, and IWV zeolites. Herein, we report on the expansion of ADOR zeolites with a new branch related to the IWR topology, which is yet unattainable experimentally but theoretically predicted as highly promising adsorbents for CO_2_ separation applications. The optimization of not only the chemical composition but also the dimensions of the crystalline domain in the parent IWR zeolite in the Assembly step was found to be the key to the success of its ADOR transformation into previously unknown IPC-17 zeolite with an intersecting 12 × 8 × 8-ring pore system. The structure of the as-prepared IPC-17 zeolite was verified by a combination of microscopic and diffraction techniques, while the results on the epichlorohydrin ring-opening with alcohols of variable sizes proved the molecular sieving ability of IPC-17 with potential application in heterogeneous catalysis. The proposed synthesis strategy may facilitate the discovery of zeolite materials that are difficult or yet impossible to achieve using a traditional bottom-up synthesis approach.

## Abstract 2

Addressing the synthesis–property relations in the Assembly step of ADOR enabled to design previously unknown IPC-17 zeolite by optimization of the chemical composition and crystallite dimensions in the parent IWR germanosilicate.

## Graphical abstract

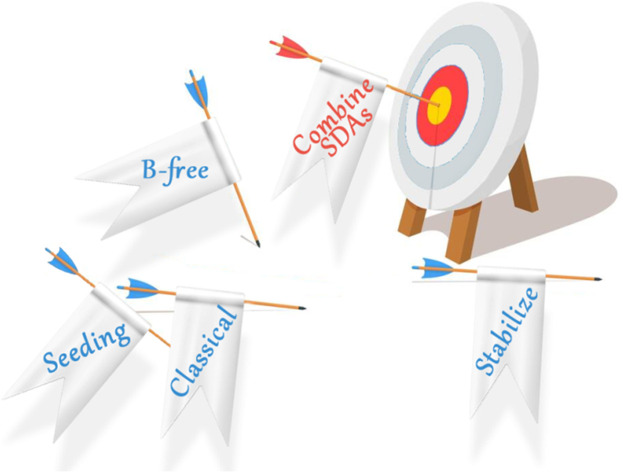

## 1 Introduction

The rational synthesis of structurally distinct zeolites remains a primary target because of the intimate relationships that exist between the functioning of these materials in the most important applications in adsorption and catalysis and their structural properties.^1^ However, conventional (primarily solvothermal) synthesis approaches suffer from a limited understanding of the underlying mechanism of a zeolite formation and thus the deliberate selection of the conditions for rational design of their structural properties.^2^ The last decade has brought about the development of an efficient post-synthesis method for the predictable design of zeolite frameworks with previously unknown topologies, as opposed to a conventional trial-and-error direct synthesis approach.^3^ This multistep Assembly-Disassembly-Organization-Reassembly strategy (ADOR) is based on the chemically selective deconstruction/reorganization of particular Ge-rich double-four-ring (D4R) structural units located in-between the intact silica layers in some germanosilicate zeolites.^4^ The beneficial feature of the ADOR synthesis approach is that it enables the prediction of the topology, approximate pore sizes, textural properties and other structure-related characteristics of potential zeolite products even before their synthesis.^5^

**Scheme 1:**
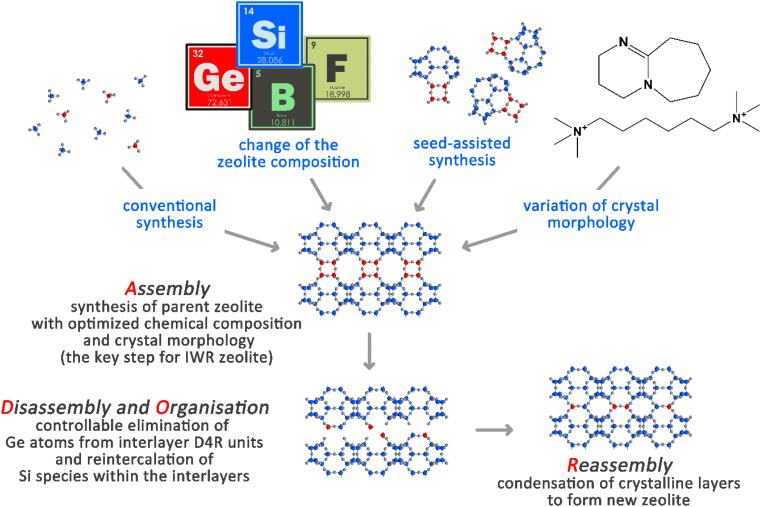
Schematic representation of the ADOR steps for the IWR zeolite under study. Ge atoms participating in the formation of D4R units in-between the crystalline silica layers are highlighted in red, while Si atoms are highlighted in blue.

The first Assembly step of ADOR is crucial for designing the features of a parent germanosilicate zeolite (usually, the chemical composition of the framework in terms of Si/Ge ratios) that are decisive for the success of further Disassembly and Reassembly steps (Scheme 1).^6^ In turn, the variation of the conditions in the Disassembly and Organization steps (pH, duration, and temperature of the treatment) has already yielded a broad array of materials that were not possible to achieve using conventional solvothermal synthesis. The materials derived from one parent germanosilicate (*e.g.*, UTL) may differ in how the material layers are linked (for example, UTL-D4R *vs.* UTL-S4R, where the double four-membered ring or single four-membered ring interlayer linker was removed from a parent zeolite^4^) or in how the material layers are placed with respect to each other (for example, UTL-D4R *vs.* layer-shifted UTL-D4R^7^).

Although many germanosilicate frameworks, such as (i) UTL, UOV, *CTH, IWW, and IWV and (ii) IWR, ITR, and ITH, are described as appropriate for ADOR transformations, the successful application of the ADOR strategy was reported only for zeolites of type (i).^8–14^ For germanosilicates from group (ii), the failure of the ADOR was generally explained by the suboptimal chemical composition of the initial zeolites to be adjusted in the Assembly step.^15^ In particular, although they were successfully disassembled under the appropriate conditions, ITR- and ITH-derived silica layers were shown to suffer during the Organization and Reassembly stages.^15^ For the same reason, the ADOR products of IWR zeolite transformation remained unattainable^15^ although predicted to be thermodynamically feasible synthetic targets.^5^

The IWR zeolite was first synthesized from B- or Al-containing germanosilicate reaction mixtures in hydroxide media using hexamethonium dihydroxide (SDA1) as a structure-directing agent.^16^ In turn, the syntheses in fluoride media allow one to obtain IWR with a wide range of Si/Ge and B/(Si + Ge) molar ratios.^17^ Similarly to other germanosilicates, Ge atoms in IWR are preferentially located in D4R units.^18^ With an increase in Ge concentration in the framework (Si/Ge = 2.5), Ge atoms start occupying the T sites in the D4Rs and layers.^17,18^ The presence of Ge or B atoms in the layers of IWR compromises their hydrolytic stability. Specifically, IWR with a high content of Ge (Si/Ge = 1.8)^19^ or B (Si/Ge = 6.9, but 14.8 mol% B)^15^ reacts with water at room temperature, resulting in a complete degradation to amorphous material. Recently, the application of specially designed organic SDAs has been reported for the preparation of Ge-free IWR zeolites containing either Al^20–22^ or B^22^ atoms in a silica framework. However, the lack of Ge atoms, which form the regioselectively located centers of hydrolytic instability, precludes the use of these materials for the top-down synthesis of IWR-derived ADOR zeolites.

Since the discovery of the ADOR strategy, optimization of the chemical composition of the parent zeolite in the Assembly step and of the conditions in the subsequent Disassembly, Organization, and Reassembly stages has become a standard approach for the successful transformation of various germanosilicates (*e.g.*, UOV,^9,23^ IWW,^13,24^ and *CTH^11^) into new crystalline microporous materials. Based on the already published results, the optimization of the chemical composition of IWR zeolite in terms of both Si/Ge and Si/B molar ratios is seen as a crucial milestone that unavoidably precedes the application of the ADOR synthesis approach to this framework type. However, to the best of our knowledge, the hydrothermal synthesis of well-known IWR zeolite was not previously considered the subject of deeper investigation in the context of its ADOR transformation. This study presents detailed research on the synthesis–property relations laid in the Assembly step of IWR germanosilicate towards designing theoretically predicted but experimentally yet unattainable ADOR zeolites. Herein, we report the optimization of the chemical composition and crystallite dimensions in the IWR as the key to the success of its ADOR transformation into the previously unknown IPC-17 zeolite. Microscopy imaging in combination with X-ray diffraction data revealed a reduced size of the interlayer units (D4Rs in IWR *vs.* S4Rs in IPC-17) at the preservation of the IWR layer within the framework of the designed zeolite. The pore system of IPC-17 zeolite is formed by intersecting 12-, 8-, and 8-ring pores, which are predicted to enable relatively strong CO_2_ adsorption.^25^ Furthermore, IPC-17 was shown to demonstrate shape-selectivity in the ring-opening of epichlorohydrin with alcohols of different sizes (*i.e.*, ethanol and iso-propanol).

## 2 Results and discussion

### 2.1 Different strategies for the Assembly of parent IWR zeolite

Because the appropriate composition of the parent framework and the conditions for its selective fragmentation have not yet been found, the IWR has remained unapplicable for the ADOR transformation. Although such a transformation was predicted to be possible,^5^ a synthesis of any ADOR derivative of IWR has been considered hardly feasible in practice owing to the poor stability of the respective zeolitic layers usually containing three-valent elements.^26^ To overcome this limitation, we applied various strategies in the Assembly step aimed at improving the hydrolytic stability of the IWR layers, while preserving the lability of the interlayer D4R units. The strategies are as follows.

**Fig. 1:**
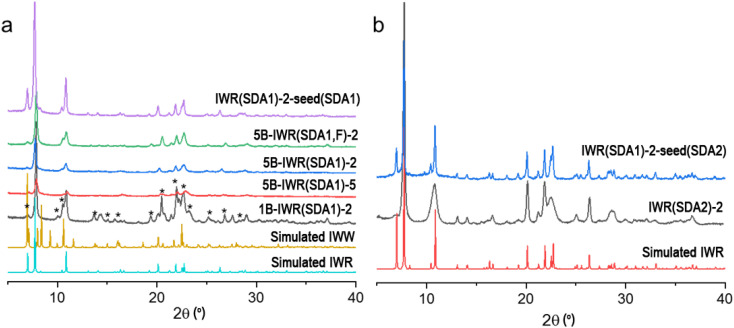
(a) XRD patterns of IWR samples prepared from reaction mixtures with variable B content and Si/Ge molar ratios in the presence of SDA1. Characteristic diffraction lines of the IWW zeolite admixture in the 1B-IWR(SDA1)-2 sample are marked by asterisks. (b) XRD patterns of IWR samples prepared from B-free reaction mixtures with a Si/Ge molar ratio of 2 using different SDAs.

(1) Variation in the Si/Ge ratio in the parent IWR framework. The Si/Ge ratio below 6 was proposed as an appropriate value to ensure a sufficient composition of labile interlayer D4R units for their reconstruction upon disassembly.^15^ To optimize the Si/Ge ratio in the IWR zeolite, we used direct SDA1-assisted synthesis^16^ in B-containing reaction mixtures while varying Si/Ge molar ratios from 2 to 5 (mB-IWR(SDA1)-*n* samples depicted in Fig. 1a).

(2) Minimization of structural defects (silanols) in the IWR framework by the Assembly of the parent zeolite in a fluorine-containing reaction mixture. Silanol defects are known as sites of hydrolytic instability in zeolites.^27^ In turn, minimization of the concentration of the silanol groups in silica and aluminosilicate zeolites by conducting hydrothermal crystallization in F^−^-containing reaction mixtures was reported to improve their hydrolytic stability.^28^ In this work, the synthesis of IWR zeolite was conducted in the presence of fluoride ions to stabilize its borosilicate layers (sample 5B-IWR(SDA1, F)-2 in Fig. 1a).

(3) Decreasing the B content in the IWR layers using the seeding method. The B-containing IWR sample 5B-IWR(SDA1)-2 prepared by applying the original method^16^ was submitted as seeds for the synthesis of B-depleted IWR zeolite from the B-free reaction mixture. The sample obtained in the first iteration of seed-assisted synthesis was used as the seed source for the second iteration under the same conditions (IWR(SDA1)-2-seed(SDA1) sample in Fig. 1a). The resulting IWR(SDA1)-2-seed(SDA1) sample contained <0.1 mol% of B according to the chemical analysis. Direct SDA1-assisted synthesis of B-depleted IWR zeolite was unsuccessful without seeding, as it led to the formation of the undesired phase (IWW zeolite) as an admixture or even as the main crystallization product (*e.g.*, sample 1B-IWR(SDA1)-2 depicted in Fig. 1a).

(4) Elimination of B atoms from IWR layers using SDA2-assisted crystallization. Originally synthesized as borogermanosilicate with hexamethonium dihydroxide as SDA, IWR was shown to be formed as germanosilicate zeolite also using 1,8-diazabicyclo[5.4.0]undec-7-ene (SDA2).^29^ The specific feature of thus prepared B-free germanosilicate IWR (sample IWR(SDA2)-2 depicted in Fig. 1b) is the negligible intensity of (001) diffraction peak at around 7° 2*θ*, corroborating with a decrease in the size of coherently scattering domains in the crystallographic direction *c.* This result agrees with the study of Wu *et al.*, who first reported the crystallization of nanolayered IWR zeolite when using SDA2 as the structure-directing agent.^29^ As axis *c* corresponds to the direction of the main structural changes expected after ADOR transformation, the decrease in crystal size along this direction was anticipated and further validated (*vide infra*) to compromise the crystallinity of the daughter zeolite formed following the Disassembly-Organization-Reassembly steps.

(5) Elimination of B atoms from the IWR layers while optimizing the crystallite dimensions. Combining the synthesis in B-free media using SDA2 with further utilization of the obtained IWR(SDA2)-2 as seeds, the SDA1-assisted synthesis was expected to allow one to avoid both the boron atom-induced hydrolytic instability of the IWR layers and the sacrificial decrease in crystallite thickness in the crystallographic direction *c.* In line with our hypothesis, the results of the PXRD for the IWR(SDA1)-2-seed(SDA2) sample showed a pronounced intensity of (001) diffraction peak at around 7° 2*θ*, while its FWHM was similar to those of the B-IWR(SDA1) samples (Fig. 1b). In addition, the results of the chemical analysis did not detect B within the quantification limit of the ICP-MS technique.

Optimization of synthesis conditions within the proposed strategies allowed us to synthesize IWR materials as crystalline single-phase solids (Fig. 1). Further Disassembly was performed *via* the hydrolysis of the prepared zeolites in 0.1–12 M HCl^30^ or using HCl vapor-assisted transformation.^12^

### 2.2 Post-synthesis modification of IWR zeolites *via* the ADOR protocol

**Scheme 2:**
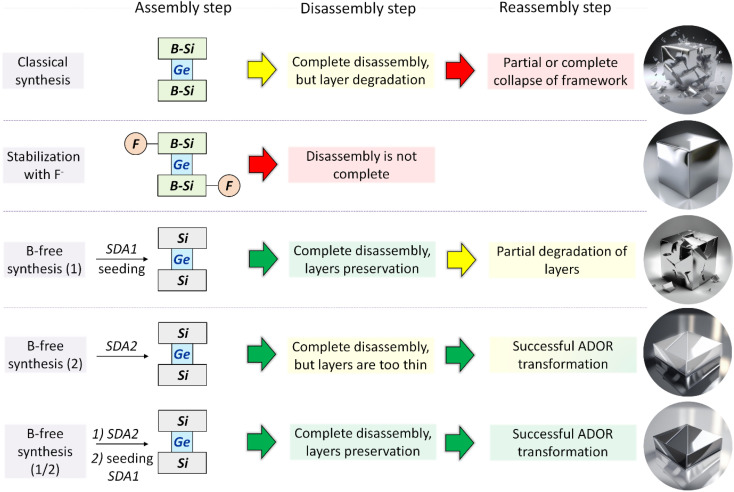
Variation in the synthesis approach in the Assembly step of IWR zeolite and its consequence for the following Disassembly and Reassembly steps of the ADOR structural transformation.

XRD was used as the key characterization technique that allowed us to judge the success of the ADOR transformation of IWR samples with tunable chemical compositions and crystalline dimensions (Fig. S1–S5†). Specifically, the right-shift value of the “interlayer” (00*l*) diffraction line (at 7° 2*θ* in the parent IWR zeolite) indicates the change in the interlayer distance upon ADOR transformation, while the intensities of the “intralayer” (*hk*0) reflections represent the crystallinity of the layers. Based on the XRD analysis, the outcomes of three types were documented upon Disassembly (under optimized conditions using 12 M HCl) and further Reassembly (*i.e.*, condensation at elevated temperature) of the prepared IWR zeolites depending on their structural characteristics. These results are summarized in Scheme 2 as follows.

**Fig. 2:**
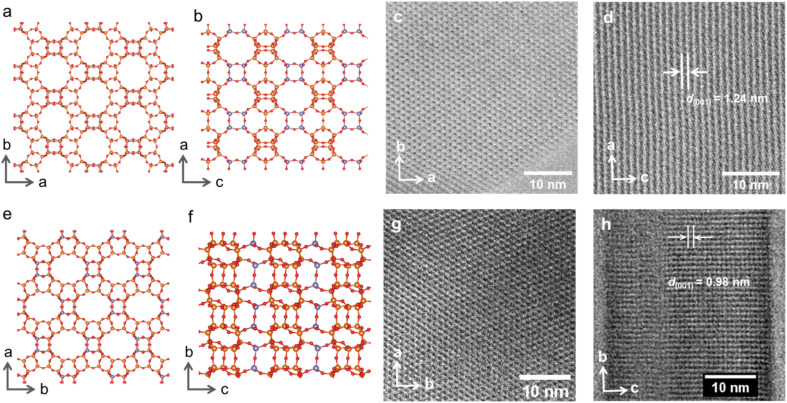
Crystallographic models of IWR zeolite in the (a) *a* × *b* (“top”) and (b) *a* × *c* (“side”) projections demonstrating the layer plane and connectivity between layers, respectively. Corresponding ABF-STEM images of the initial IWR in the (c) *a* × *b* and (d) *a* × *c* projections. Crystallographic models of IPC-17 zeolite in the (e) *a* × *b* (“top”) and (f) *b* × *c* (“side”) projections showing the maintenance of the layer structure but a change in the “interlayer” connectivity. ABF-STEM images of IPC-17 zeolite corresponding to *a* × *b* (g) and *b* × *c* (h) projections. T atoms in the D4Rs and S4Rs are highlighted with a dark grey color in the models.

(1) The right-shift of (00*l*) “interlayer” reflection value, which was smaller than the theoretically predicted one^5^ (*e.g.*, 2*θ* position for (001) peak < 8.8°), suggested unsuccessful Disassembly (red arrows in Scheme 2). This outcome was observed for the 5B-IWR(SDA1, F)-2 material, in which the fluorine-stabilized IWR layers were not completely separated (Fig. S1†). This result may be explained by the stabilization of not only borosilicate layers but also Ge-D4R units with fluoride ions, typically located at or close to the center of the D4R units in various zeolites.^31–34^ In addition to failure in the Disassembly of 5B-IWR(SDA1, F)-2, the amorphization of the material at the Reassembly step may suggest the insufficient stabilization of boron-containing IWR layers with fluoride ions. Similar to 5B-IWR(SDA1, F)-2, full collapse of the zeolite framework was observed after Disassembly-Organization-Reassembly steps for the B-rich samples 5B-IWR(SDA1)-2 and 5B-IWR(SDA1)-5 prepared using the classical SDA-1-assisted method (Fig. S1†).

(2) The right-shift of (00*l*) “interlayer” reflection to 2*θ* position at 8.8°, which corresponded to the theoretical prediction for the IWR-S4R daughter,^5^ was observed for both B-depleted IWR(SDA1)-2-seed(SDA1) (Fig. S1†) and B-free IWR(SDA2)-2 (Fig. S2, a†). Acid treatments of these samples did not alter the structural integrity of the IWR layers (as suggested by the preservation of “intralayer” (*hk*0) diffraction lines after the Disassembly step, Fig. S1 and S2†), although the crystallinity of the material (designated as IPC-17) formed in the Reassembly step was relatively low (*vide infra*). This result, shown in Scheme 2 as a yellow arrow, was considered partial success. Based on the characteristics of the IWR zeolites used as parent materials, different origins of the low crystallinity for IPC-17 samples are anticipated: (1) the presence of boron traces (<0.1 mol% according to ICP-MS analysis) in IWR(SDA1)-2-seed(SDA1), which may limit the hydrolytic resistance of the crystalline layers; (2) the low thickness of the crystalline domains in *c* direction previously reported for IWR(SDA2) materials and verified in IWR(SDA2)-2 sample using XRD analysis (Fig. 1), which may cause a partial disorder of the layers condensed in the Reassembly step.

(3) The application of the ADOR method to the B-free IWR(SDA1)-2-seed(SDA2) obtained using a sequential approach comprising (i) direct synthesis of B-free IWR seeds in the presence of SDA2, followed by (ii) seed-assisted crystallization in the presence of SDA1 results in IPC-17 (its structure is compared to the parent IWR in Fig. 2) with the highest crystallinity among the prepared IPC-17 samples (Fig. S2, b†). The key to this successful structural transformation (shown as a green arrow in Scheme 2) lies in optimizing the chemical (Si/B = ∞) and structural characteristics (sufficient crystallite thickness in the crystallographic direction *c*) of the parent IWR zeolite in the Assembly step. The subsequent Disassembly and Reassembly steps do not significantly affect the morphology of the zeolite crystals, as indicated by SEM images for starting IWR and final IPC-17 (Fig. S3†).

The conditions applied in the Disassembly step, such as the acidity of the solution, were also found to be decisive for the IWR-to-IPC-17 transformation. For example, when IWR(SDA1)-2-seed(SDA2) was treated with 0.1 M HCl aqueous solution, the “interlayer” (001) diffraction line shifted only to around 7.2° 2*θ*, indicating the incomplete hydrolysis of “interlayer” linkages (Fig. S4†). In contrast, the formation of a phase-pure IPC-17 zeolite was observed upon the Reassembly of IWR(SDA1)-2-seed(SDA2) hydrolyzed using a 12 M HCl aqueous solution or VPT with HCl vapor (Fig. S4†).

### 2.3 Structural characteristics of IWR-derived IPC-17 zeolite

Compared to the IWR framework, which can be viewed as silica/borosilicate crystalline layers connected by D4R units, IPC-17 presents S4R “interlayer” connections (Fig. 2a). The participation of “intralayer” atoms in the formation of IPC-17 “interlayer” linkages is consistent with the mobility of Si species at low pH.^30^ As a result of the removal and/or replacement of Ge with Si, the chemical composition of the zeolite framework changes significantly after the IWR-to-IPC-17 transformation (Si/Ge = 3.7 and 15 for IWR and IPC-17, respectively).

**Fig. 3:**
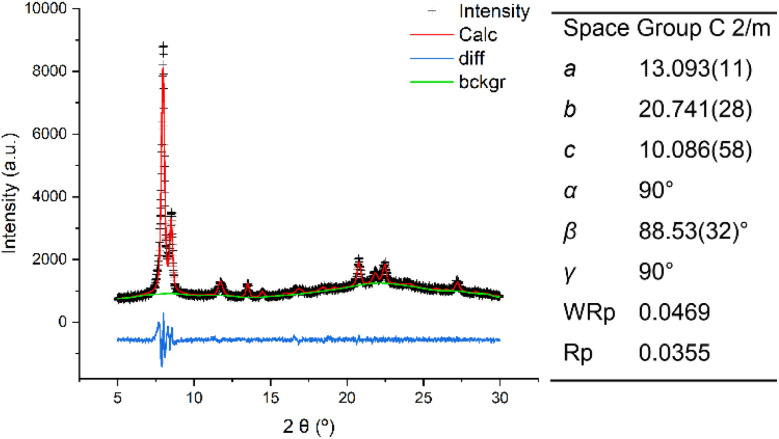
XRD patterns and unit cell parameters of IPC-17: experimental (black), calculated after Le Bail refinement (red), and the difference between them (blue).

Based on the topology of the initial IWR zeolite, several possible structures of “IWR-S4R” were predicted.^35^ The particular type of the structure of “IWR-S4R” (*i.e.*, IPC-17) was initially identified by comparing the experimental XRD pattern with those predicted computationally for different unit cell symmetries. The unit cell of IPC-17 was confirmed by a structureless Le Bail refinement against the experimentally obtained diffraction data using the GSAS package (Fig. 3). Despite our efforts, a more convincing refinement of the structure of IPC-17 using the Rietveld method was impossible. Applying short acquisition times did not allow one to obtain suitable diffraction patterns using either XRD or RED, while the increase in the acquisition time led to the collapse of the material.

However, the successful formation of the new zeolite IPC-17 was also confirmed by the results of high-resolution transmission electron microscopy (HRTEM) (Fig. 2). A good match of the “top” view of HRTEM images was found in accordance with the model (Fig. 2c and g) considering the negligible difference in the topology of layers in the parent IWR and daughter IPC-17 zeolites (Fig. 2a and e, *a* × *b* projections for both materials are practically the same). Furthermore, the decrease in d-spacing along the *c* direction from 1.24 to 0.98 nm after the IWR-to-IPC-17 transformation correlates with the value predicted for the IPC-17 model and corresponds to the change in the interlayer distance caused by the replacement of the D4R by the S4R units (Fig. 2b, f, d and h, “side” view). In turn, a reasonably good match of the respective P2 symmetry-averaged ADF-STEM image of IPC-17 with the crystallographic model of IPC-17 zeolite in the *b* × *c* (“side”) projection (Fig. S5†) agrees with the expected transformation of the “interlayer” pores after ADOR.

**Fig. 4:**
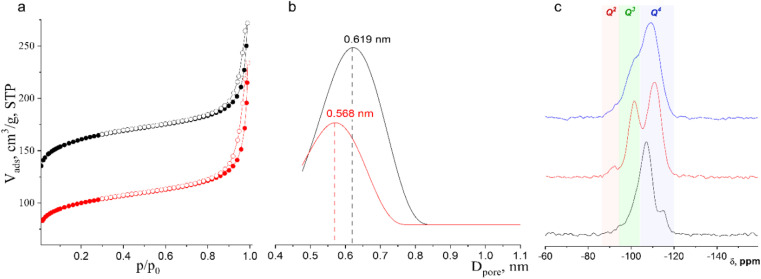
(a) Argon adsorption/desorption isotherms for parent IWR and daughter IPC-17 zeolites; (b) pore size distribution of parent IWR (black) and daughter IPC-17 zeolites (red); and (c) ^29^Si MAS NMR spectra of initial IWR (black), intermediate IPC-17P (red), and IPC-17 (blue) samples.

Ar physisorption revealed the anticipated change in the pore size distribution after IWR-to-IPC-17 transformation (Fig. 4). The parent IWR zeolite has a three-dimensional channel system formed by interconnected 12- (along 001 projection) and 10- (along 010 and 001 directions) ring pores. During the IWR-to-IPC-17 transformation, 10-ring channels become 8-ring ones owing to the shrinkage of interlayer connecting units, while 12-ring channels are preserved. This change in the pore system naturally led to a decrease in the micropore volume and a change in the average pore size. The volume of micropores in IPC-17 was found to be lower in the starting zeolite (0.150 *vs.* 0.086 cm^3^ g^−1^ for IWR *vs.* IPC-17, respectively). Importantly, the shape of the isotherm did not change significantly after the transformation from IWR to IPC-17 (Fig. 4a), indicating that no new pores (such as mesopores) were formed owing to the partial degradation of the framework. Because of the presence of pores of various sizes and their interconnectivity in both parent and daughter zeolites, the differentiation of individual pores in these materials is challenging. However, the average (apparent) pore size reflecting the change in the size of individual channels can still be estimated using pore-size distribution plots (Fig. 4b), which show a decrease in the average channel diameter from 0.619 nm for IWR to 0.566 nm for IPC-17.

The change in structural characteristics caused by the IWR-to-IPC-17 transformation, particularly the average pore size, has the potential to be used for sorption/catalysis applications. Despite the use of zeolites as solid acid catalysts usually suggesting direct or post-synthesis incorporation of three or four valent elements, such as Al, Ti, Sn, and Zr, into the framework positions, the use of the model reaction catalysed by Ge-associated active sites allows one to prove the concept with IPC-17 germanosilicate. Thus, the ring-opening of non-symmetric epoxides, such as epichlorohydrin (1-chloro-2,3-epoxypropane), with alcohols of different sizes (such as ethanol and iso-propanol)^36,37^ was studied over parent IWR and daughter IPC-17 zeolites. Notably, the distribution between two alcoholysis products that contain ethoxy- or isopropoxy-groups (Fig. S6†) differed for IWR (EtO-/i-PrO- ratio is 1.0–1.1) and IPC-17 (1.5–1.7) when using an equimolar mixture of both alcohols. This result is consistent with a decrease in the average pore size of IPC-17 *vs.* IWR reflected in the dissimilar molecular sieving ability of these zeolites and suggested the shape-selective performance of IPC-17 zeolite in the selected reaction.

The evolution of Si environments upon the Disassembly and Reassembly of IWR(SDA1)-2-seed(SDA2) germanosilicate was studied using ^29^Si MAS NMR spectroscopy. The fully condensed framework of IWR contains mainly Q^4^ Si atoms corresponding to the Si(OT)_4_ fragments (the signals at −107 and −115 ppm shown in Fig. 4c are assigned to the Si(OSi)_4_ and Si(OSi)_3_(OGe), respectively) and only a small fraction of Q^3^ atoms corresponding to the silanol defects (the signal at −101 ppm assigned to Si(OH)(OSi)_3_ fragments). The IWR-to-IPC-17 rearrangement leads to the evolution of the relative amount of deficient Q^3^ Si species and fully condensed Q^4^ atoms. First, treatment with HCl vapors results in the formation of a large fraction of silanols (signal at −101 ppm) as remnants after the decomposition of Ge-rich “interlayer” D4R units. At this stage, the material can be considered the layered precursor of IPC-17 zeolite (IPC-17P). This metastable IPC-17P intermediate has a high concentration of reactive hydroxyls on the surface and is characterized by their regular location as the removal of D4R connections leaves the ordered pattern of surface hydroxyls from the Si–O–Ge linkages. Notably, the signal at −92 ppm corresponding to Q^2^ Si atoms (Si(OSi)_2_(OH)_2_) detected in disassembled IPC-17P can be related to further hydrolysis of Q^3^ Si sites proceeding in parallel with the aforementioned Q^4^ → Q^3^ transformation. Following the calcination of IPC-17P leads to a decrease in the signal intensity attributed to silanol groups (Q^3^), reflecting the condensation of silanols (Si–OH + HO–Si → Si–O–Si) and the formation of an IPC-17 zeolite with a dominant fraction of Q^4^ atoms. The difference in the positions of the Q^4^ peaks in the starting and final zeolites can be related to the change in the chemical composition and thus the environment of the Si atoms at the framework positions surrounded either by *x*Si + (4 − *x*)Ge (in IWR) or mostly by 4Si (in IPC-17).

## 3 Conclusions

By manipulating germanosilicate zeolites with hydrolytically unstable Ge–O bonds regioselectively located in a framework, the top-down ADOR synthesis strategy has enabled the preparation of diverse zeolites, which are thus far unattainable using conventional bottom-up synthesis approaches. Considering the structural diversity of germanosilicates that can be subjected to ADOR transformation, this synthetic strategy was expected to significantly expand the number of functional zeolite materials. However, our incomplete understanding of the synthesis–property relations of the parent germanosilicates (such as IWR zeolite) laid in the Assembly step restricted the discovery of ADOR materials, which were theoretically predicted as feasible synthetic targets.

This study has addressed the synthesis–property relations in the Assembly of IWR zeolite and designed the parent germanosilicate to be appropriate for ADOR transformation. Using fluorine-assisted crystallization, modification of the organic structure-directing agent, seeding, and their combinations, the chemical composition and crystallite dimensions of IWR zeolite were varied in the Assembly step, while the optimization of these characteristics was found to be crucial for the ADOR transformation of IWR into the previously unknown IPC-17 zeolite. While demonstrating how the chemical and structural characteristics of a parent zeolite can be designed in the first step of the ADOR sequence and how these characteristics affect the behavior of a zeolite during the Disassembly and Reassembly steps, this study provides the first experimental evidence on the importance of the dimensions of crystalline domains in the parent germanosilicate for the outcome of ADOR. Therefore, the reported findings can help to engineer further potential families of ADOR zeolites and to expand this synthesis approach beyond traditional germanosilicate parents with a unidirectional location of Ge-enriched D4R units.

The pore system of the IPC-17 zeolite is formed by intersecting 12-, 8-, and 8-ring pores, which are promising for applications in adsorption and shape-selective catalysis. In this regard, based on the results of our recent theoretical investigation,^25^ IPC-17 is suggested as a potential adsorbent of CO_2_. In turn, the catalytic performance of IPC-17 in epichlorohydrin ring-opening with ethanol and iso-propanol convincingly evidenced the shape-selective behavior of this zeolite in the Ge Lewis acid-catalysed reaction. However, isomorphous substitution combined with the recovery and recycling of rarely abundant germanium^38^ is considered a viable way to use the unique structural characteristics of IPC-17 in different applications. The development of Sn- and Zr-substituted IPC-17 zeolites with variable Lewis acid centers, which are active sites for the redox reactions of carbonyl compounds, is currently in progress.

## 4 Experimental methods

### 4.1 Synthesis of hexamethonium dihydroxide (SDA1)

SDA1 was prepared using a method described elsewhere.^39^ For the preparation of hexamethylene-bis(trimethylammonium) dibromide, 37.4 g of 1,6-dibromohexane (96%, Sigma Aldrich) was mixed with 82.5 g of trimethylamine solution (31–35 wt% in ethanol, Sigma Aldrich) and 200 ml of absolute ethanol (Lachner) with a magnetic stirrer for 2 days at ambient temperature. After that, the reaction mixture was washed with ethyl acetate (99.97%, Fisher Scientific) and diethyl ether (99.97%, Lachner). The final product was separated by filtration and dried at room temperature for 12 h.

Hexamethylene-bis(trimethylammonium) dibromide was transformed into hydroxide form using Ambersep® 900(OH) anion exchange resin (Acros Organics, 0.8 mmol of SDA1 per 1 g of anion exchange resin). The solution of SDA1 was concentrated under low pressure (35 Torr) at 30 °C until the hydroxide concentration >1.0 M.

### 4.2 Assembly of IWR zeolite

#### 4.2.1 SDA1-assisted crystallization of B-IWR(SDA1,F) and B-IWR(SDA1) samples

The synthesis of boron-containing IWR zeolites was performed according to ref. 16 in the presence of SDA1 (and HF). The starting gel had the following molar composition: (1 − *x*) SiO_2_: *x* GeO_2_: (0.01–0.1) BO_1.5_: 0.225 SDA1: *y* HF: 5H_2_O, where *x* = 0.33 or 0.17, *y* = 0 or 0.1.

Typically, boric acid (>99.5%, Sigma-Aldrich) and germanium oxide (99.99%, Sigma-Aldrich) were dissolved in a 1.0 M SDA1 solution. TEOS (98%, Aldrich) was then added, and the mixture was gently stirred at room temperature until complete evaporation of the alcohol formed. After that, 49 wt% solution of hydrofluoric acid (Sigma Aldrich) was added. The resulting gel was autoclaved at 175 °C under tumbling (∼60 rpm) for 10 days. The obtained solid was separated by filtration, washed with distilled water, and dried overnight at 95 °C. The occluded hexamethonium was removed from the samples by thermal treatment at 300 °C for 3 h and then at 580 °C for 3 h. The heating rate was 1 °C min^−1^. The samples were designated as mB-IWR(SDA1,F)-*n* for the materials synthesized in the presence of HF or mB-IWR(SDA1)-*n* for the materials synthesized without HF; *m* and *n* values represent mol% of B and Si/Ge molar ratios in the reaction mixture, respectively.

#### 4.2.2 Seed-assisted crystallization of B-depleted IWR(SDA1)-seed(SDA1)

The 5B-IWR(SDA1)-2 sample was used as seeds for the synthesis of the IWR zeolite in a boron-free reaction mixture. The preparation of the reaction mixture and hydrothermal crystallization were performed similarly to those described for the 5B-IWR(SDA1)-2 sample but without adding boric acid while replacing 10 wt% of silica sources with zeolite seeds. The prepared zeolite was calcined and used as the seed source for the second iteration of seed-assisted crystallization under the same conditions. The respective B-depleted sample was denoted as IWR(SDA1)-2-seed(SDA1).

#### 4.2.3 SDA2-assisted crystallization of the B-free IWR(SDA2) sample

The IWR(SDA2) sample was synthesized according to ref. 29 using 1,8-diazabicyclo[5.4.0]undec-7-ene (SDA2) (98%, Sigma-Aldrich) as SDA with the molar composition of the reaction mixture as follows: 1.0 SiO_2_: 0.5 GeO_2_: 1.5 SDA2: 7H_2_O. In a typical synthesis, 3.8 g SDA2 was first added to 6.3 ml of water, followed by the dissolution of 2.6 g GeO_2_ in the mixture. 3 g of fumed silica was then added to the pellucid solution, and the mixture was stirred for 30 min. The synthesis mixture was crystallized at 170 °C in a Teflon-lined stainless-steel autoclave under static conditions for 7 days. Once the crystallization was finished, the product was collected by filtration, washed with distilled water and then dried at 60 °C overnight. To remove SDA2, the sample was calcined at 550 °C for 6 h at a heating rate of 1 °C min^−1^ under air flow and denoted as IWR(SDA2)-2.

#### 4.2.4 SDA-1-assisted crystallization of the B-free IWR(SDA1)-seed(SDA2) sample using IWR(SDA2)-2 as seeds

The boron-free IWR(SDA1)-2-seed(SDA2) sample was synthesized in the presence of SDA1 using IWR(SDA2)-2 as seeds. Synthesis was carried out using gels with the following chemical composition: 0.66 SiO_2_: 0.33 GeO_2_: 0.225 SDA1: 5H_2_O. Typically, 1.7 g GeO_2_ was dissolved in the HMI solution; then, 7.0 g tetraethyl orthosilicate was slowly added to the solution. The reaction mixture was stirred in an open plastic beaker to evaporate excess water and ethanol until the desired composition was achieved. Then, 300 mg of IWR(SDA2)-2 was added. The gel was transferred to a Teflon-lined stainless-steel autoclave and crystallized under tumbling at 175 °C for 10 days. The resulting product was collected by filtration, washed with deionized water, and dried at 60 °C overnight. To remove the SDA, the sample was calcined at 550 °C for 6 h at a heating rate of 1 °C min^−1^ under air flow.

### 4.3 Post-synthesis treatment of IWR zeolites

Following the classic ADOR protocol,^30^ 0.1 g of calcined IWR sample was treated with 10 ml 0.1 M or 12 M HCl (Sigma-Aldrich) at room temperature for 6 h. Solid products were recovered by centrifugation or filtration, washed thoroughly with methanol (99.98%, Lachner) and acetone (99.99%, Lachner), and dried at 60 °C. The obtained solids were calcined at 450 °C for 2 h with a temperature ramp of 1 °C min^−1^.

The IWR samples were also subjected to vapour-phase-transport (VPT) treatment according to the procedure reported in ref. 12 For that, 0.1 g of calcined IWR sample was placed on the polytetrafluoroethylene (PTFE) membrane over 10 ml of 12 M hydrochloric acid solution at 25 °C for *τ* = 16 h.

### 4.4 Characterisation

The structure and crystallinity of the zeolite samples under study were determined by powder X-ray diffraction (XRD) using a Bruker AXS-D8 Advance diffractometer outfitted with a LYNXEYE XE-T detector using CuK_α_ radiation in Bragg–Brentano geometry at a scan rate of 0.25 2*θ* min^−1^.

The chemical compositions of the zeolite samples were determined by performing ICP-MS analysis (ThermoScientific iCAP 7000). For that, 50 mg of zeolite was dissolved in a mixture of 2 ml of 48% HF, 4 ml of 67% HNO_3_, and 4 ml of 36% HCl in a microwave. After cooling, the HF excess was eliminated by complexation with 15 ml of a saturated solution of H_3_BO_3_, and the final mixture was again treated in the microwave. Thereafter, the solutions under analysis were collected and diluted with ultrapure water to a total volume of 250 ml.

Argon adsorption/desorption isotherms were measured using an ASAP 2020 (Micromeritics) static volumetric apparatus at −186 °C. Prior to the sorption measurements, all samples were degassed using a Micromeritics Smart Vac Prep instrument at 300 °C for 8 h. The NLDFT algorithm using standard Micromeritics software for cylindrical pores was applied to estimate micropore volumes (*V*_mic_) and pore size distributions based on the adsorption branch of the argon isotherms.

The solid-state ^29^Si MAS NMR spectra were recorded using an Agilent DD2 500WB spectrometer at resonance frequencies of 99.30 MHz. All MAS NMR measurements were performed using a commercial 3.2 mm triple resonance MAS probe. ^29^Si chemical shifts were referenced to tetramethylsilane (TMS) at 0 ppm using a deshielding scale. Saturation combs were applied prior to all repetition delays. The ^29^Si 1D experiments were performed at a sample spinning frequency of 10 kHz using a pulse length of 3 μs and a recycle delay of 60 s. During the ^29^Si acquisition period, proton broadband decoupling was applied with a continuous wave sequence using a nutation frequency of 100 kHz. 1000 scans were acquired for each ^29^Si NMR spectrum.

Scanning electron microscopy (SEM) was used to study the size and shape of zeolite crystals without coating them with any metals (SEM, Quanta 200F).

Scanning transmission electron microscopy (STEM) measurements were performed using a JEOL JEM NEOARM-200F microscope equipped with a Schottky-type field emission gun (FEG) and a TVIPS XF416 CMOS camera. The samples were prepared by the direct deposition of crystals on a holey carbon supported on a 300-square mesh copper TEM grid. Owing to the beam sensitivity of the sample, low electron-dose conditions were used. Alignment of the microscope was done using a standard sample of gold nanoparticles. STEM images were recorded simultaneously in both the ADF and ABF modes. The probe size was 0.1 nm, and the dwell time was 4 μs per pixel. The images were registered using a condenser lens aperture of 40 microns (convergence angle 29 mrad), and the ADF collection angle ranged from 27 to 110 mrad. Selected ABF- and ADF-STEM images were post-processed by filtering using the average background subtraction filter (ABSF) with Gatan Digital Micrograph software. The P2 symmetry averaging (using CRISP 2.2 software) was applied to the ADF-STEM image of the “side” view of IPC-17. The resulting image is shown in Fig. S5.†

### 4.5 Catalytic tests

Ring-opening of the epichlorohydrin with alcohols over IWR(SDA1)-2-seed(SDA2) and IPC-17 zeolites was carried out in 25 ml round-bottom glass batch reactors using a Star-Fish multi-experiment workstation (Radleys Discovery Technologies). Prior to the test, the catalysts were activated at 450 °C for 4 h at a rate of 5 °C min^−1^. A gas chromatograph (Agilent 7890B) fitted with a non-polar HP-5 column (length 30 m, diameter 0.320 mm, and film thickness 0.25 m) and a flame ionization detector were used to analyze samples taken at regular intervals throughout the reaction. Using a Thermo Scientific ISQ LTTRACE 1310 GC/MS, the reaction products were identified. The amounts of reactant and product were evaluated using internal standard calibration.

The reaction mixture was prepared by mixing 3 mmol of epoxide, 5 ml of alcohol (*e.g.*, ethanol, iso-propanol or 1/1 mol mol^−1^ mixture of both), and 0.83 mmol of mesitylene (internal standard). The mixture was heated to 70 °C under vigorous agitation with a magnetic stirrer, and 0.05 g of catalyst was added.

The conversion (*X*), yield (*Y*), and selectivity (*S*) values were calculated using the following equations:

1 *X* (%) = [(*n*(reactant)_0_ − *n*(reactant)_*t*_)/*n*(reactant)_0_] × 100,

2 *Y* (%) = [*n*(product)_*t*_/*n*(reactant)_0_] × 100,

3 *S* (%) = [*Y*/*X*] × 100,

where *n*(reactant) and *n*(product) values in eqn (1) and (2) were determined using the internal standard calibration method, with mesitylene (Sigma Aldrich, 98%) as the internal standard and commercially available epichlorohydrin (VWR Chemicals, 99%), 1-chloro-3-ethoxy-2-propanol (Sigma Aldrich) and 1-chloro-3-isopropoxy-2-propanol (Sigma Aldrich). Only terminal ethers (1-chloro-3-ethoxy-2-propanol and 1-chloro-3-isopropoxy-2-propanol) were found among the products of the reaction studied, while terminal alcohols (1-chloro-2-ethoxy-3-propanol and 1-chloro-2-isopropoxy-3-propanol) were not formed under the applied conditions.

## Author contributions

Q. Y.: Investigation, formal analysis (synthesis, post-synthesis, XRD measurement), writing – original draft; V. K.: investigation, formal analysis (synthesis, post-synthesis, XRD and NMR measurement); M. M.: investigation, formal analysis (TEM, SEM); S. A.: investigation, formal analysis (synthesis, post-synthesis, catalytic tests); Y. Z.: investigation (supplementary post-synthesis, XRD); P. S. W.: investigation, formal analysis (XRD, Le Bail refinement). R. E. M.: Funding acquisition, writing – review & editing; J. Č.: supervision, funding acquisition, writing – review & editing; M. S.: supervision, project administration, funding acquisition, writing – review & editing. M. O.: Conceptualization; supervision; writing – review & editing. All authors contributed to the final version of the manuscript.

## Conflicts of interest

There are no conflicts to declare.

## Supplementary Material

TA-012-D3TA06161B-s001
