# Supplementary material for: ADOR zeolite with 12 × 8 × 8-ring pores derived from IWR germanosilicate
Source: J Mater Chem A Mater. 2023 Nov 29;12(2):802–12. doi: 10.1039/d3ta06161b (PMC10763919; doi:10.1039/d3ta06161b)
Supplement: TA-012-D3TA06161B-s001 [file TA-012-D3TA06161B-s001.pdf]

Electronic Supplementary Material (ESI) for Journal of Materials Chemistry A.
This journal is © The Royal Society of Chemistry 2023

Supporting Information

ADOR zeolite with 12×8×8-ring pores derived from IWR germanosilicate

Qiudi Yue,[†a] Valeryia Kasneryk,[†a] Michal Mazur,[a] Sarra Abdi, [a] Yong Zhou,[a] Paul S. Wheatley,[b] Russell E. Morris,[ab] Jiří Čejka,[a] Mariya Shamzhy*[a] and Maksym Opanasenko*[a]

[a]*Department of Physical and Macromolecular Chemistry, Faculty of Science, Charles University, Hlavova 8, 128 43 Prague 2, Czech Republic*

[b]*EaStCHEM School of Chemistry, University of St Andrews, St Andrews KY16 9ST UK.*

† These authors contributed equally

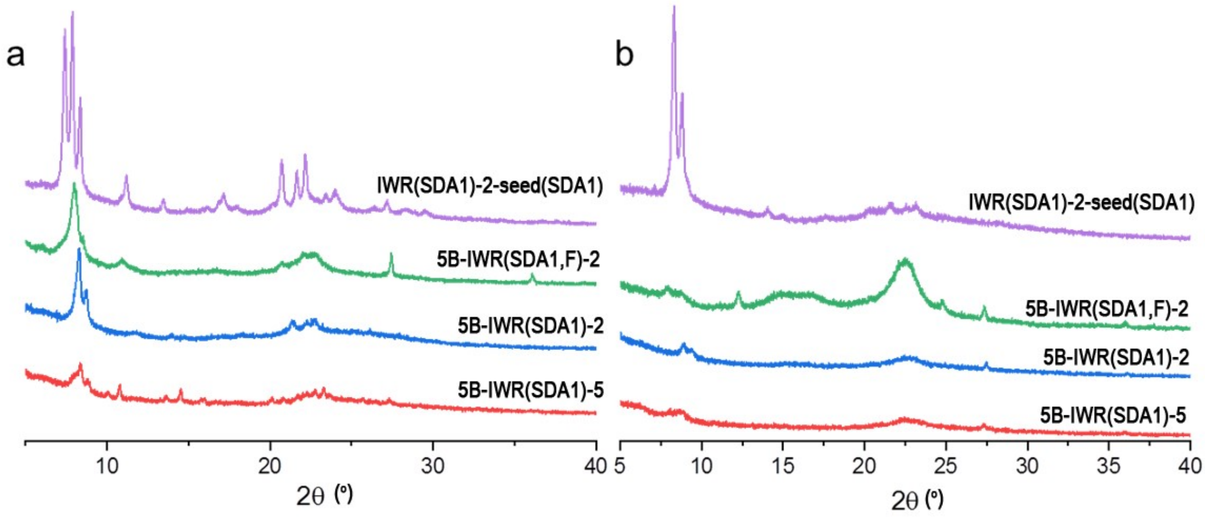

Fig S1. XRD patterns of IWR samples treated with 12 M HCl solution (a) and subsequently calcined (b).

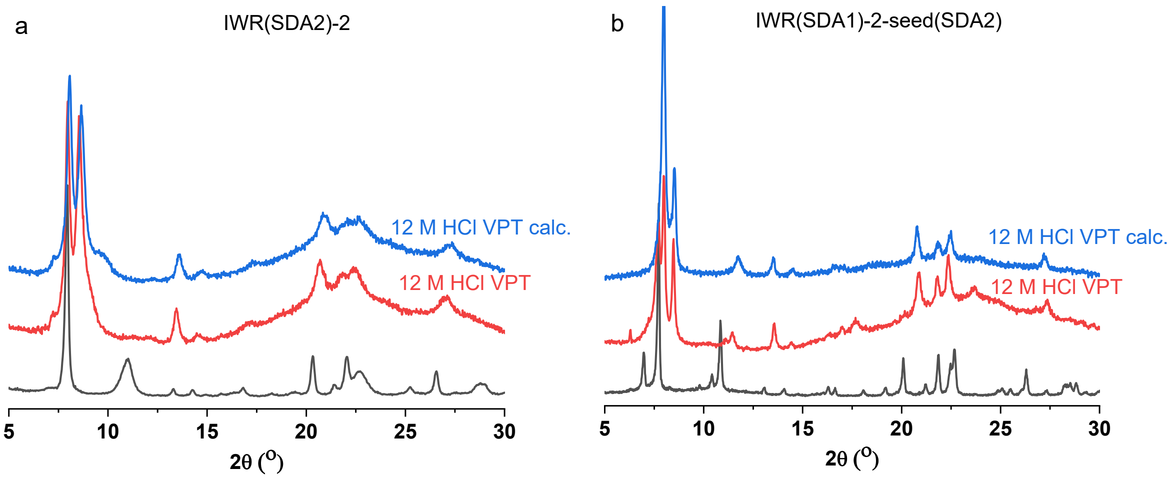

Fig. S2 XRD patterns of IWR zeolite (black) treated with the vapours of 12 M HCl under VPT conditions before and after calcination: (a) IWR(SDA2)-2 and (b) IWR(SDA1)-seed(SDA2).

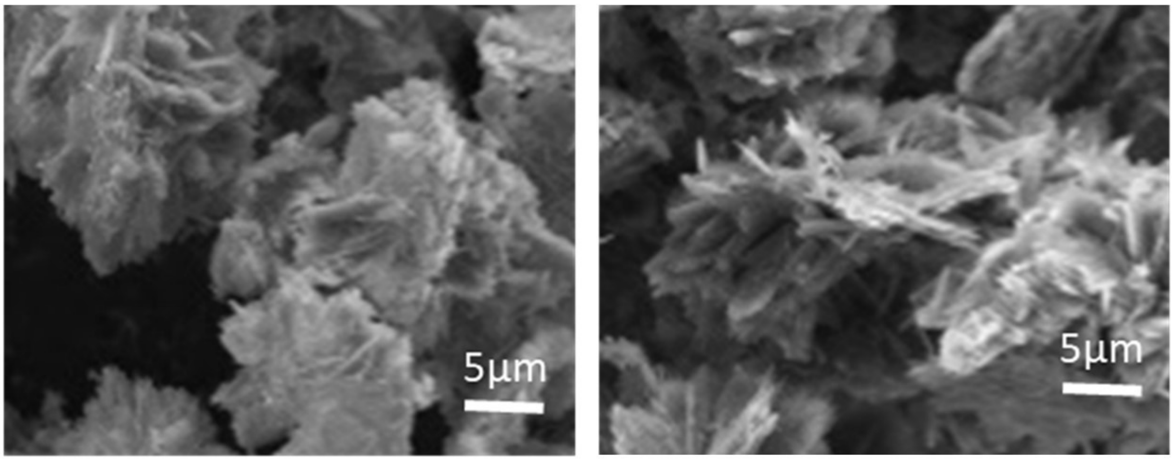

Fig. S3 SEM images of IWR(SDA1)-2-seed(SDA2) zeolite sample (left) and the daughter IPC-17 zeolite (right).

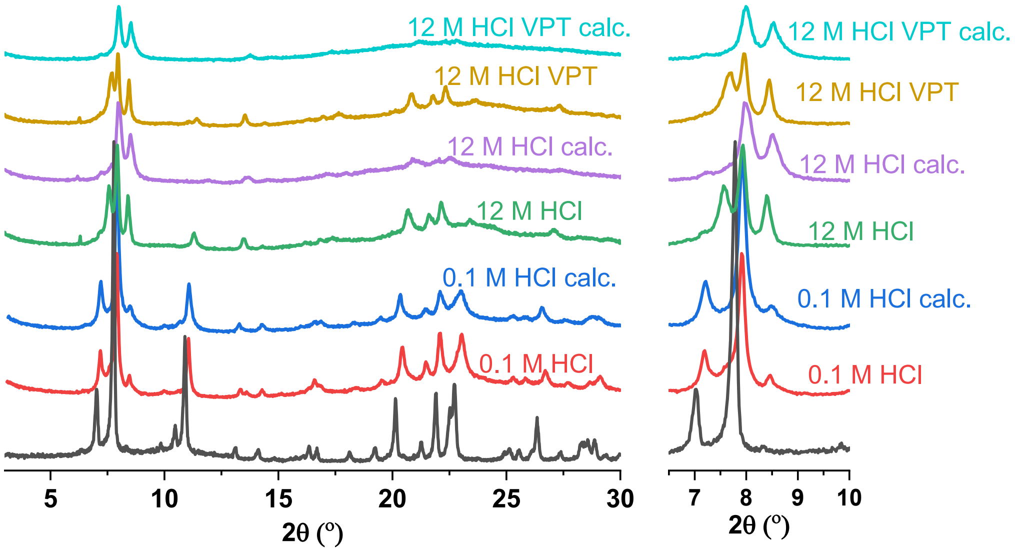

Fig. S4. XRD patterns of IWR(SDA1)-2-seed(SDA2) sample (black) treated with 0.1 M HCl, 12 M HCl, and 12 M HCl vapours under VPT conditions before and after calcination. The incomplete disassembly in 0.1 M HCl is revealed by the small right-shift of (011) diffraction line at 7° 2θ for + 0.2° 2θ.

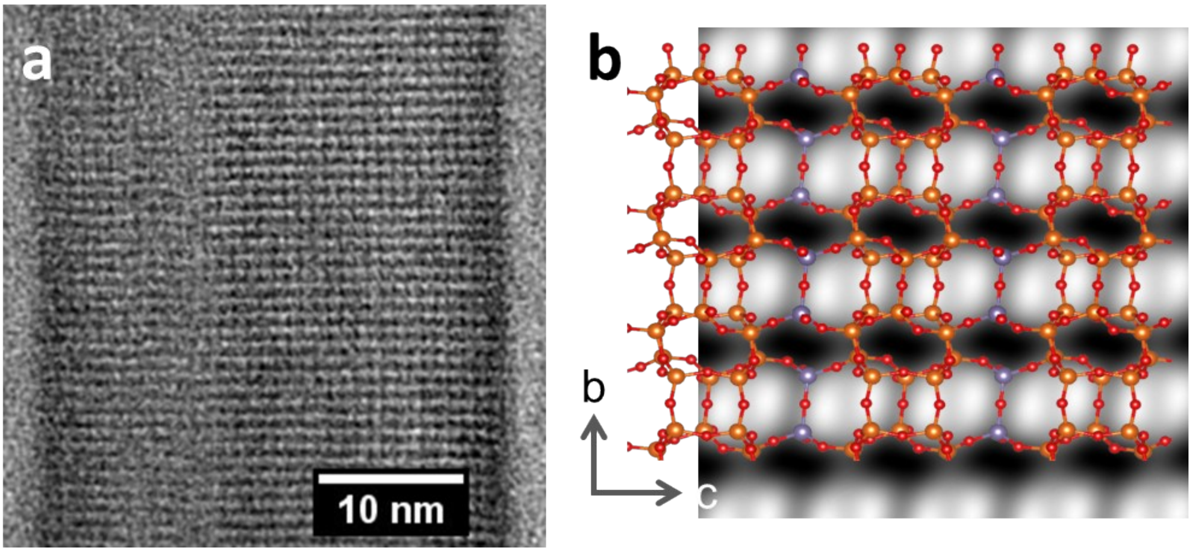

Fig. S5. ABSF filtered ABF-STEM image of IPC-17 zeolite corresponding to b×c projection (a) and overlay of the respective P2 symmetry-averaged ADF-STEM image with the crystallographic model of IPC-17 zeolite in the same projection (b).

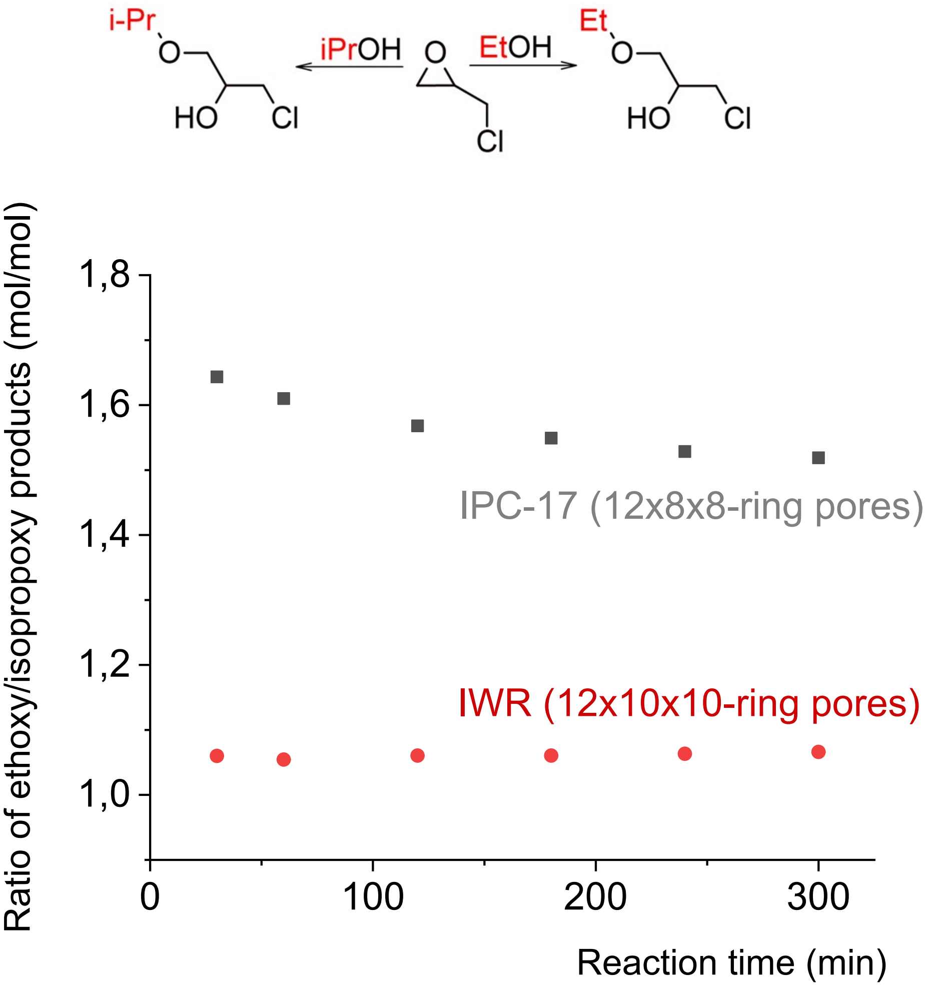

Fig. S6. Epichlorohydrin ring-opening with alcohols under study (top). Molar ratio of the epichlorohydrin ring-opening products formed over parent IWR zeolite and daughter IPC-17 zeolite when using equimolar mixture of ethanol and *iso*-propanol (bottom).
